# A new hybodontiform shark (*Strophodus* Agassiz 1838) from the Lower Cretaceous (Valanginian-Hauterivian) of Colombia

**DOI:** 10.7717/peerj.13496

**Published:** 2022-06-02

**Authors:** Jorge D. Carrillo-Briceño, Edwin-Alberto Cadena

**Affiliations:** 1 Paläontologisches Institut und Museum, Universität Zürich, Zürich, Switzerland; 2 Facultad de Ciencias Naturales, Grupo de Paleontología Neotropical Tradicional y Molecular (PaleoNeo), Universidad del Rosario, Bogotá, Colombia; 3 Smithsonian Tropical Research Institute, Panama City, Panama

**Keywords:** Gondwana, South America, Rosa Blanca Formation, Acrodontidae, Durophagous, Zapatoca

## Abstract

The vertebrate marine faunas that inhabited northern South America during the Cretaceous are still poorly known. This study is a contribution to a growing wave of new studies on Lower Cretaceous vertebrates from Colombia. Here we report and describe a new species of a hybodontiform shark of the genus *Strophodus*, which we named *Strophodus rebecae* sp. nov., based on isolated teeth, that were collected in Valanginian-Hauterivian rocks of the Rosa Blanca Formation (Carrizal and El Sapo Members) near the town of Zapatoca, Santander Department, Andes of Colombia. In addition, we describe two other fragmented teeth assigned to *Strophodus* sp. from the Rosa Blanca Fm. The new species from Colombia represents the only Cretaceous record of *Strophodus* from Gondwana, offering new insights into the paleogeographic distribution of the genus, as well as increasing the knowledge about the scarce hybodontiform paleodiversity known from South America. The presence of *Strophodus* in the Rosa Blanca Formation suggests that these durophagous (shell-crushing) fishes played an important role as predators of the abundant and diverse invertebrate fauna present in these ancient tropical coastal ecosystems of Gondwana.

## Introduction

Hybodontiform sharks were one of the dominant and most successful freshwater and marine chondrichthyan lineages during the Triassic and Jurassic, but their abundance and diversity began to decline during the Middle Jurassic, to finally become extinct at the end of the Cretaceous (Kriwet & Benton, 2004; Rees, 2008; Rees & Underwood, 2008; Cappetta, 2012; Cuny, 2013). The fossil record of hybodontiform sharks mostly represents teeth, cephalic and fin spines, and dermal denticles (*e.g.*, Cappetta, 2012); although some articulated and delicately preserved specimens have also been reported (Stumpf et al., 2021, and references therein). Many hybodontiform species were described using isolated teeth and spines only, one example is the genus *Asteracanthus* (Agassiz, 1837), considered to be one of the most common hybodontiforms, given that teeth and spines traditionally referred to this genus have been reported worldwide from Middle Triassic to Upper Cretaceous strata (Rees & Underwood, 2008; Cappetta, 2012; Szabó & Főzy, 2020; Stumpf et al., 2021; Stumpf, Meng & Kriwet, 2022). Agassiz (1837) named the type species *Asteracanthus ornatissimus* using isolated fin spines characterized by prominent stellate tubercles from the Upper Jurassic of England. Using some ornamental variations as differential characters, Agassiz (1837) also erected the species *Asteracanthus acutus*, *Asteracanthus minor* and *Asteracanthus semisulcatus*. This laid the foundation for the subsequent naming of many new species based only on isolated fin spines, whose validity has been ambiguous in many cases, particularly due to the lack of discrete morphological characters for use in species differentiation (see Stumpf et al., 2021). Later, Agassiz (1838) created the genus *Strophodus*, based on distinctive crushing-type teeth from the Jurassic of Europe. The discovery of isolated fin spines of *Asteracanthus* associated with *Strophodus* teeth in Jurassic sediments of England led Woodward (1889) to consider the latter genus as a junior synonym of *Asteracanthus*, a taxonomic scheme that has remained unquestioned for more than one century. Recently, Stumpf et al. (2021) reported a well-preserved and articulated hybodontiform skeleton from the Late Jurassic of Germany with a characteristic combination of tuberculate dorsal fin spines, characteristic of *Asteracanthus*, and multicuspid teeth (with resemblance to *Hybodus* Agassiz, 1837 and *Egertonodus* Maisey, 1987) that markedly differ from crushing-type teeth referred previously to this genus. This evidence led Stumpf et al. (2021) to propose *Asteracanthus* and *Strophodus* as two valid genera, which can be readily distinguished from each other by differences in both teeth and dorsal fin spine morphology. For more details about historical and taxonomic background, fossil record and diversity of *Asteracanthus,* see Stumpf et al. (2021).

**Figure 1:**
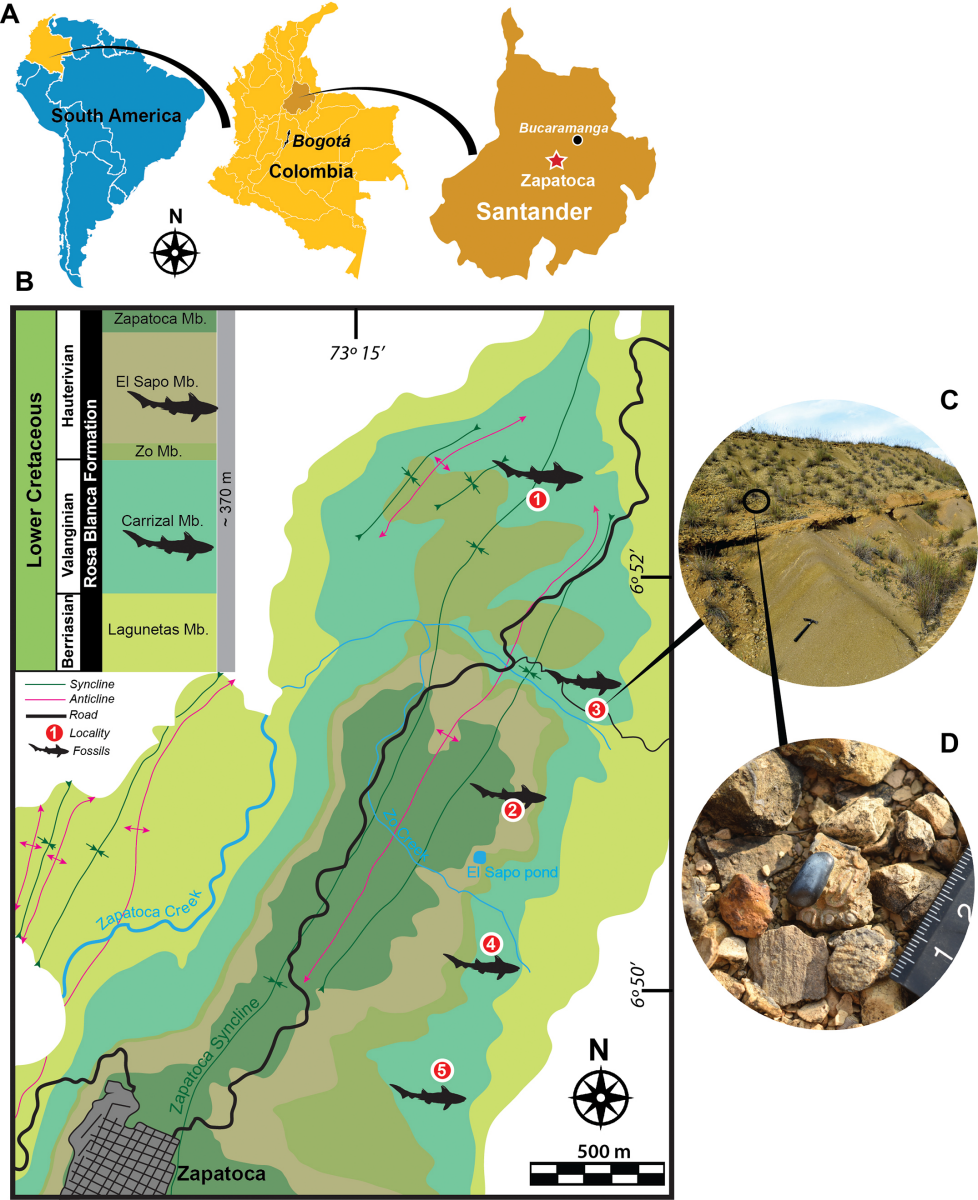
Geographical location, geological map and localities. (A) Geographical location. (B) Geological map of fossiliferous localities studies here of the Rosa Blanca Formation modified after Etayo-Serna & Guzmán-Ospitia (2019). (C–D) Outcrop from Pico de la Vieja South locality, showing some isolated dental pycnodontiform fish remains. Fossiliferous localities: (1) La Virgen West, (2) El Sapo North, (3) Pico de la Vieja South, (4) El Sapo South, (5) El Caucho.

The present contribution focuses on the description of a new species of *Strophodus* based on isolated teeth from the Rosa Blanca Formation (Fm.) (Valanginian-Hauterivian), Andes of Colombia (Figs. 1A–1B). The new material described herein represents the first Cretaceous record of the genus *Strophodus* from Gondwana. The new data shed additional light onto the scarce hybodontiform paleodiversity known from South America, which is represented by few reports from the Triassic and Jurassic of Argentina (Cione et al., 2002; Johns, Albanesi & Voldman, 2014), Upper Jurassic and Lower Cretaceous of Brazil (Pinheiro et al., 2013, and references therein), Upper Jurassic-?Lower Cretaceous of Uruguay (Soto, Perea & Toriño, 2012), and Lower Cretaceous of Colombia (Carrillo-Briceño et al., 2016). Additionally, two fragmented teeth assigned to *Strophodus* sp. from the Rosa Blanca Fm. are also described here.

## Material & Methods

### Fossil collection

Most of the fossils described here were found isolated on the surface of biomicrites (wackestone) layers (Figs. 1C–1D) and very few of them appeared *in situ* inside the rocks. Although hybodontiforms occur along the entire sequence of the Carrizal and El Sapo Members of the Rosa Blanca Formation, they are particularly more abundant between layers (P^I^ to Y) of the Carrizal Member and between layers (A to J) of El Sapo Member, following the stratigraphic identification of Etayo-Serna & Guzmán-Ospitia (2019). Field permits were obtained from the Servicio Geológico Colombiano, permit (No 20203800077871).

### Fossil material

The fossil material described here belongs to the Paleontological Collection of the Facultad de Ciencias Naturales, Universidad del Rosario, Bogotá, Colombia. Most of the fossils were found relatively clean, for some of them rock matrix was carefully removed using a pneumatic air scribe. To preserve the fossils’ integrity and original preservation we avoided applying any type of epoxy resins or consolidants.

### Localities

Hybodontiform teeth were collected in five localities from two members of the Rosa Blanca Fm. (Fig. 1B). From the Carrizal Member, the following localities have been sampled: Pico de la Vieja South (6°51′19, 53″N, 73°13′57, 13″W), La Virgen West (6°52′24,61″N, 73°14′18,35″W), El Sapo South (6°′24,61″N, 73°14′18,35″W), and the El Caucho (6°50′16,95″N, 73°14′56,82″W). The Carrizal Member corresponds to a shallow (∼10 m depth) marine rock sequence, with abundant hardground *Thalassinoides* beds and decimetric wackestone beds alternating with calcareous mudstones; the total thickness is approximately 110 m; the age of this member, based on ammonoids (*Thurmanniceras pertransiens* and *Saynoceras verrucosum*), is Lower Valanginian-Upper Valanginian (Etayo-Serna & Guzmán-Ospitia, 2019). Only one specimen was sampled in the El Sapo North locality from the El Sapo member (6°50′43, 08″N, 73°14′29, 79″W). El Sapo Member is also a shallow marine sequence made of conspicuous *Thalassinoides* beds and decimetric wackestone alternating with terrigenous calcareous mudstone that indicate a major input of continental sediments in contrast to the Carrizal Member; the total thickness of the El Sapo member is approximately 80 m; the age for El Sapo Member based also on ammonoids (*Shasticrioceras anglicum*, *Bochianites kiliani, Oosterella colombiana* and *Olcostephanus boussingaultii*) is Lower Hauterivian (Etayo-Serna & Guzmán-Ospitia, 2019).

### Comparisons

Taxonomic identification is based on an extensive bibliographical review and anatomical comparison with fossil specimens housed in the following Swiss collections: Natural History Museum of Basel (NMB); Palaeontological Institute and Museum at the University of Zurich (PIMUZ); JURASSICA Museum (MJSN) in Porrentruy, and René Kindlimann (RK) private collection with public access, Uster. Here we follow the proposal of Stumpf et al. (2021) to distinguish *Asteracanthus* and *Strophodus* as two valid genera. Systematic placement follows Cappetta (2012) and Stumpf et al. (2021). Tooth descriptive terminology follows that of Rees & Underwood (2008), Leuzinger et al. (2017), Szabó & Főzy (2020), and Kumar et al. (2022).

### Nomenclatural act

The electronic version of this article in Portable Document Format (PDF) will represent a published work according to the International Commission on Zoological Nomenclature (ICZN), and hence the new names contained in the electronic version are effectively published under that Code from the electronic edition alone. This published work and the nomenclatural acts it contains have been registered in ZooBank, the online registration system for the ICZN. The ZooBank LSIDs (Life Science Identifiers) can be accessed and the associated information viewed through any standard web browser by appending the LSID to the prefix http://zoobank.org/. The LSID for this publication is: urn:lsid:zoobank.org:pub:11ED37F2-6168-41CB-8DF3-AAE56976D9D6. The online version of this work is archived and available from the following digital repositories: PeerJ, PubMed Central SCIE and CLOCKSS.

## Results

### Systematic paleontology

**Table:** 

Class Chondrichthyes Huxley, 1880
Subclass Elasmobranchii Bonaparte, 1838
Order †Hybodontiformes Patterson, 1966
Family †Acrodontidae Casier, 1959
Genus †*Strophodus*Agassiz, 1838
Type species †*Strophodus longidens*Agassiz, 1838

*Strophodus* recognized species.—Based on the list presented by Stumpf, Meng & Kriwet (2022) and the species recognized by Szabó & Főzy (2020), Kumar et al. (2022), and Sharma & Singh (2021), *Strophodus* is represented by at least 12 species, which in stratigraphic order include: (1) *S. cf. reticulatus* Agassiz, 1838 from the Middle Triassic of Switzerland (see Rieppel, 1981) and *S. reticulatus* from the Bathonian–Tithonian of England, France, Germany, Hungary and Switzerland (see Stumpf, Meng & Kriwet, 2022 and references therin); (2) *S. smithwoodwardi* (Peyer, 1946) from the Toarcian of Switzerland; (3) *S. dunaii* (Szabó & Főzy (2020), from the Aalenian of Hungary; (4) *S. tenuis* Agassiz, 1838 from Aalenian–Bathonian strata of Germany and England (Rees & Underwood, 2008); (5) *S. longidens* (Agassiz, 1838) (type species) from the Bathonian of France; (6) *S. magnus* (Agassiz, 1838) from the Bathonian of England, France and India (Rees & Underwood, 2008; Sharma & Singh, 2021; Rigal & Cuny, 2016); (7) *S. indicus* (Sharma & Singh, 2021) from the Bathonian of India; (8) *S. jaisalmerensis* (Kumar et al., 2022) from the Bathonian of India; (9) *S. medius* (Owen, 1869) from the Bathonian–Callovian of France, England and India (Rees & Underwood, 2008; Sharma & Singh, 2021); (10) *S*. *subreticulatus* (Agassiz, 1838) from the Kimmeridgian of Switzerland; (11) *S*. *udulfensis* (Leuzinger et al., 2017) from the Kimmeridgian of Switzerland and possibly England (Stumpf, Meng & Kriwet, 2022); and (12) *S*. *tridentinus* (Zittel, 1870), from the Tithonian of Italy (considered as nomen dubium by Szabó & Főzy, 2020).

**Table:** 

**Figure 2:**
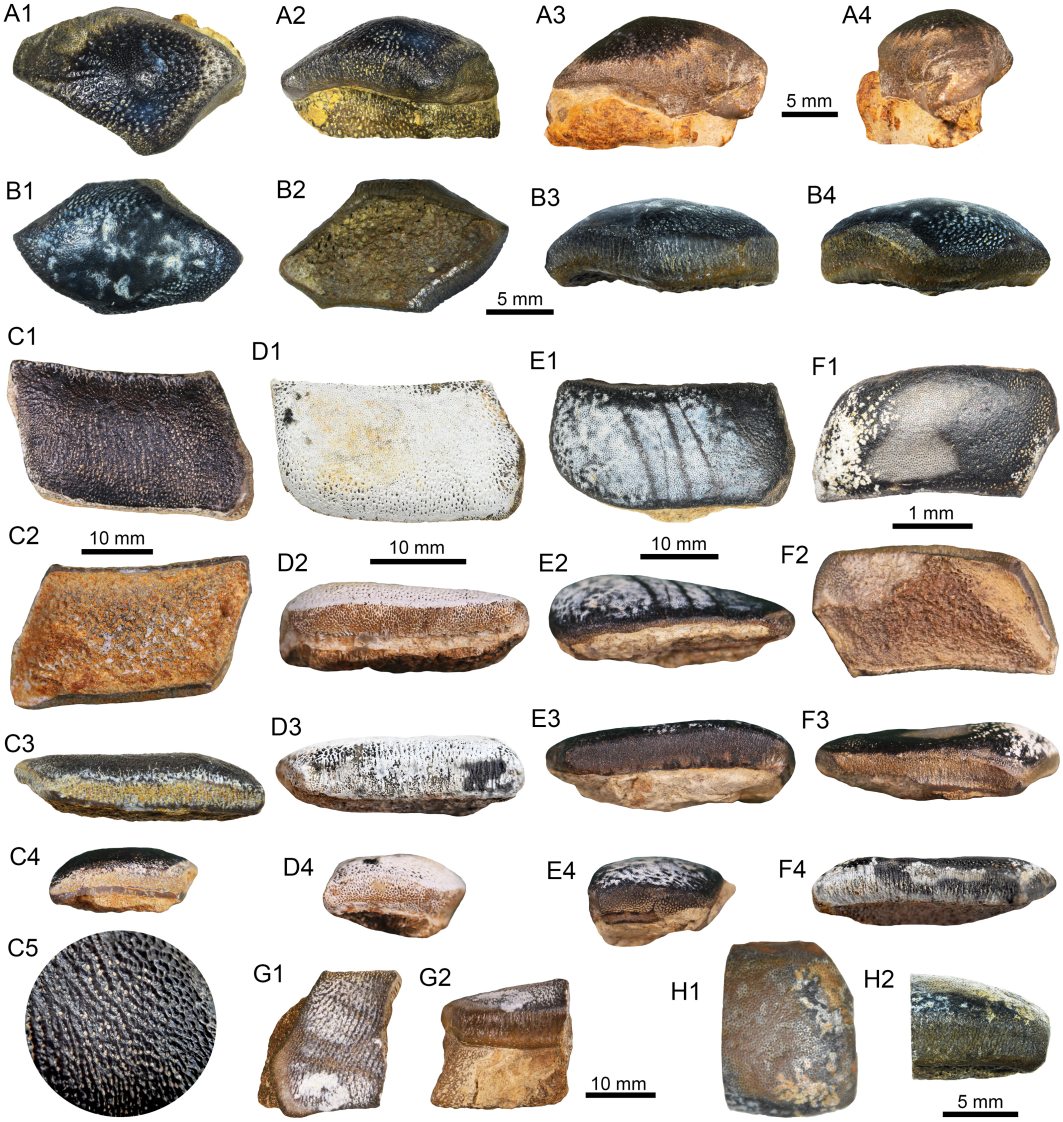
Teeth of *Strophodu* s *rebecae* sp. nov. and *Strophodus* sp. from the Rosa Blanca Formation. (A1–B4) Anterolateral teeth (A1–A4): UR-CP-0129; (B1–B4): UR-CP-0130). (C1–F4). Lateral teeth (C1–C5): holotype UR-CP-0131; (D1–D4): UR-CP-0136; (E1–E4): UR-CP-0135; (F1–F4): UR-CP-0133). (C5). Close-up of the occlusal ornamentation in the holotype UR-CP-0131. (G1–H2). Fragmented lateral teeth of Strophodus sp. (G1–G2): UR-CP-0134; H1–H2: UR-CP-0132). View: basal (B2, C2, F2,) distal (A4), labial (A3, D2, E2, F3, G2), lingual (C3, D3, E3, F4, H2), occlusal (A1, B1, C1, D1, E1, F1, C5, G1, H1), mesial (C4, D4, E4), mesio-labial (B4), mesio-lingual (A2, B3).

†*Strophodus rebecae* sp. nov.
(Figs. 2A1–2F4)

Etymology.—In honor of Rebeca Rueda, who charmingly has embraced different generations of geologists and paleontologists during fieldwork in Zapatoca.

Holotype.—UR-CP-0131 lateral tooth of indeterminate upper/lower jaw position (Figs. 2C1–2C5).

Type locality and horizon.—La Virgen West locality of the Carrizal Member, Rosa Blanca Fm. (Fig. 1B).

Referred material.—Five isolated teeth of indeterminate upper/lower jaw position. The sample includes two anterolateral teeth UR-CP-0129 (Figs. 2AI–2A4) and UR-CP-0130 (Figs. 2B1–2B4), and three lateral teeth UR-CP-0133 (Figs. 2F1–2F4); UR-CP-0135 (Figs. 2E1–2E4); UR-CP-0136 (Figs. 2D1–2D4). Differences between upper and lower dentition are poorly known in *Strophodus*, therefore distinguishing upper and lower teeth, especially using isolated elements, is a difficult task and is not supported.

Localities.—The specimens were collected from five localities (Fig. 1) of the Rosa Blanca Fm.: Carrizal Member (Pico de la Vieja South (UR-CP-0129), La Virgen West (UR-CP-0131), El Sapo South (UR-CP-0133), and El Caucho (UR-CP-0135, UR-CP-0136)), and the El Sapo Member (El Sapo North (UR-CP-0130)).

*Diagnosis*.—Species characterized by a typical crushing-type dentition with the following features: (1) Anterolateral teeth with a domed crown and a sub-rhomboidal shape in occlusal outline with vertical edges around their outline ornamented with irregular vertical ridges; (2) Relatively short lateral teeth (in comparison with other Jurassic species) with a parallelogram shape in occlusal outline, slightly domed in mesial section, with a pointed and lingually oriented mesial extremity, and labial and lingual vertical edges around their outline ornamented with irregular vertical ridges; (3) Anterolateral and lateral teeth with an occlusal ornamentation characterized by a reticulated pattern finely-pitted and without a crest.

*Description*.—The two anterolateral teeth UR-CP-0129 (13.84 mm length, 9.20 mm width) and UR-CP-0130 (12.06 mm length, 11.3 mm width) are elongated and have sub-rhomboidal shape in occlusal outline (Figs. 2A1–2B4). The crown is domed more towards its central part, and the occlusal ornamentation consist of a reticulated pattern finely-pitted, which is present from the top of dome to the upper limit of the edges. A weak crest in the mesial section of the crown and lingually displaced is visible only in UR-CP-0129 (Fig. 2A1), which may possibly reflect a sexual or positional variation between the lower and upper jaws, without ruling out that the absence of this character in UR-CP-0130 could also be due the result of functional wear. The crown is separated from the root by a shallow groove, overhanging it by vertical edges around the outline (edge angle approximately 90° with respect to the occlusal surface) which are ornamented with irregular vertical ridges. The root is preserved only in UR-CP-0129, part of this is still embedded in the matrix, and it seems to have double the height of the crown; some perforations (foramina) of varying size can be observed.

Lateral teeth are represented by the holotype UR-CP-0131 (26.94 mm length, 16 mm width, Figs. 2C1–2C5), and UR-CP-0133 (31.82 mm length, 18.2 mm width), UR-CP-0135 (32.72 mm length, 15.13 mm width), and UR-CP-0136 (28.46 mm length, 16.02 mm width), all with well-preserved crowns but no roots (Figs. 2D1–2F4). All the lateral teeth are larger than the anterolateral ones described above, and a parallelogram shape in occlusal outline characterizes them. The crown has labial and lingual vertical edges (edge angle approximately 90° with respect to the occlusal surface) parallel to each other, although the latter are slightly more concave. A sharp pointed and lingually oriented mesial extremity is present. The mesial part becomes slightly domed and no crest is visible. Although the root is not preserved in any of the lateral teeth, based on the preserved root of the anterolateral tooth UR-CP-0129 (Figs. 2A2–2A3) and the fragmented lateral tooth referred below as *Strophodus* sp. (Figs. 2G1–2G2), it is evident that the crown was overhanging the root by vertical edges which, in most cases, are ornamented with irregular vertical ridges. As in anterolateral teeth, the occlusal ornamentation consists of a reticulated pattern finely-pitted, and which is present from the top of the domed area to the upper limit of the edges (Fig. 2C5). In the holotype UR-CP-0131, the ornamentation is well preserved, while it shows signs of functional wear in other specimens.

**Figure 3:**
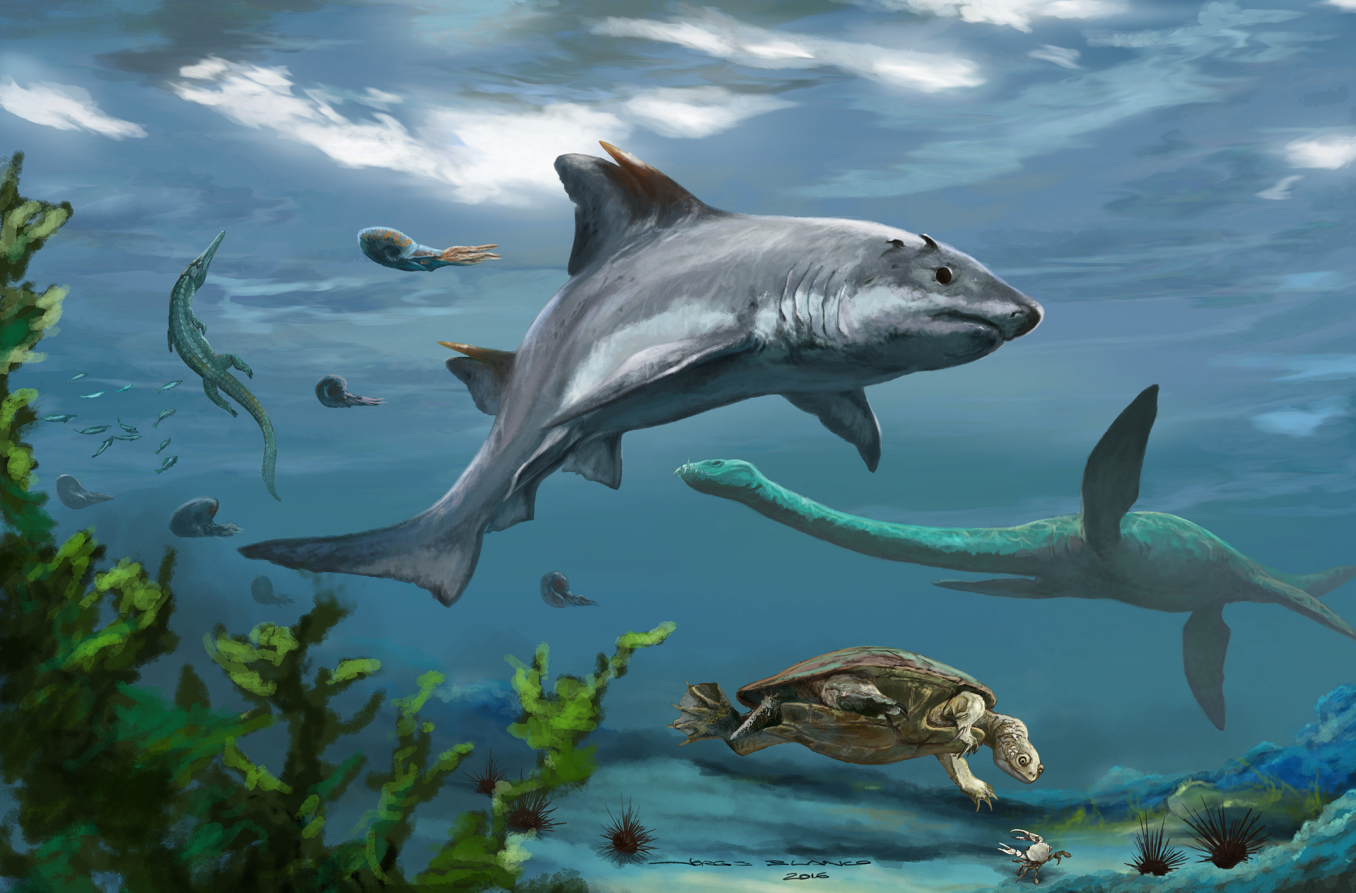
Life reconstruction of *Strophodus rebecae* sp. nov. The marine paleoenvironment of the Rosa Blanca Formation during the Valanginian-Hauterivian. Art illustration of the shark by Jorge Blanco.

*Differential diagnosis*.—Following Stumpf et al. (2021), species based on teeth and previously assigned to the genus *Asteracanthus*, but possibly referable to *Strophodus* are considered here for comparisons. Assuming this, we compare our specimens from Colombia with the at least 12 recognized *Strophodus* species from the Jurassic of Europe and India (Rees & Underwood, 2008; Szabó & Főzy, 2020; Kumar et al., 2022; Sharma & Singh, 2021; Stumpf, Meng & Kriwet, 2022). In this regard, our new species from the Lower Cretacaous of Colombia can be distinguished from the following European and Asian species by the differences presented below. (1) *Strophodus rebecae* sp. nov. differs from *S. reticulatus* from the Middle Triassic and Middle Jurassic of Europe (regarded as invalid junior synonyms of *A. ornatissimus* by Woodward (1889), see Stumpf et al. (2021)), in having thinner and mesio-distally less elongate anterolateral and lateral teeth lacking an occlusal crest with a strong ornamentation characterized by radiating ridges (Agassiz, 1838, plate 17; Stumpf et al., 2021, figs. 7S–7U). The specimen referred by Rieppel (1981) to *S*. cf. *reticulatus* from the Middle Triassic of Switzerland appears to be readily differentiated from all other *Strophodus* species by possessing a higher number of anterior tooth files (S Stumpf, pers. comm., 2022). (2) *Strophodus rebecae* sp. nov. differs from *S. dunaii* from the Aalenian of Hungary by having less rectangular lateral teeth in occlusal outline, lacking the strong ornamentation of branching ridges and the low and wide labially oriented transverse ridge that characterize *S*. *dunaii* (see Szabó & Főzy, 2020, figs. 2–3). (3) *Strophodus rebecae* sp. nov. can be distinguished from *S. tenuis* from the Aalenian–Bathonian strata of England and southern Germany mainly by the presence, in the latter, of mesio-distally more elongate and more slender anterolateral and lateral teeth with a sigmoid curvature in occlusal outline and well developed and asymmetrically situated domed areas (see Agassiz, 1838, plate 18, figs. 16–25). (4) *Strophodus rebecae* sp. nov. differs from *S*. *longidens* from the Bathonian of northern France, mainly for having mesio-distally less elongate anterolateral and lateral teeth; information on the teeth ornamentation was not described for the *S. longidens* holotype, which was destroyed during the Second World War (see Szabó & Főzy, 2020). (5) *Strophodus rebecae* sp. nov. differs from *S. magnus* from the Bathonian of England, France and India by having clearly lateral teeth that are mesio-distally less elongate (see Agassiz, 1838, tab. 18, figs. 11–15; Rees & Underwood, 2008, plate 5, figs. 1–11; Rigal & Cuny, 2016, fig. 1; Sharma & Singh, 2021, figs. 5–6). The reticulate and finely-pitted ornament pattern observed in the new species from Colombia resembles the ornamentation of *S*. *magnus*. (6) *Strophodus rebecae* sp. nov. differs from *S*. *indicus* from the Bathonian of India by having mesio-distally less elongated lateral teeth and the lack of an ornamentation characterized by enameloid folds forming ridge and groove structure (see Sharma & Singh, 2021). (7) *Strophodus rebecae* sp. nov. can be distinguished from *S*. *jaisalmerensis* from the Bathonian of India by having mesio-distally less elongated anterolateral and lateral teeth, especially the lateral ones having a more concave lingual side and a sharper-pointed and lingually oriented mesial extremity. (8) *Strophodus rebecae* sp. nov. differs from *S. medius* from the Bathonian–Callovian of England, France, and India (see Rees & Underwood, 2008; Sharma & Singh, 2021) by having less mesio-distally elongated anterolateral and lateral teeth, and a less convex and domed crown in lateral teeth that becomes flat in its most distal part. (9) If *Strophodus subreticulatus* from the Kimmeridgian of Switzerland is considered a valid species (because Woodward (1889) also regarded it erroneously as a junior synonym of *A. ornatissimus* (see Stumpf et al., 2021)), it can be differentiated of *S. rebecae* sp. nov., by the mesio-distally less elongate lateral teeth with more rectilinear edges in occlusal outline (see Agassiz, 1838, tab. 18, figs. 5–10). (10) *Strophodus rebecae* sp. nov. differs from *S*. *udulfensis* from the Kimmeridgian of Switzerland and possibly England (Stumpf, Meng & Kriwet, 2022), by having less mesio-distally elongated anterolateral and lateral teeth with less convex and domed crown and lacking the strong reticulated ornamentation that characterize *S*. *udulfensis*. (11) *Strophodus rebecae* sp. nov. can be differentiated from *S*. *tridentinus* from the Tithonian of Italy (considered as nomen dubium by Szabó & Főzy, 2020) by having lateral teeth with a less rectangular outline, a more projected and lingually oriented mesial extremity, and the lack of an occlusal crest rising a complex system of diverging folds (see Szabó & Főzy, 2020). (12) The closest tooth morphology to *Strophodus rebecae* sp. nov., was noticed in *S*. *smithwoodwardi* from the Toarcian of Switzerland. Anterolateral and lateral teeth in both species are relatively similar in occlusal outline and thickness of the crown. Nevertheless, lateral teeth in our specimens from Colombia lack the ornamentation pattern present in *S*. *smithwoodwardi* (see Peyer, 1946, plate 2–3), for which it should not be ruled out that this condition could be the result of functional wear. In the new species from Colombia both labial and lingual marginal edges tend to be vertical and well developed around the tooth outline and ornamented with irregular vertical ridges, while in all the specimens of *S*. *smithwoodwardi* that we have compared, the labial edge of the crown tends to be angled (acute angle) in profile view with a characteristic smooth surface. Like in *Strophodus rebecae* sp. nov., lingual edges *S*. *smithwoodwardi* tend to be vertical, well developed and ornamented with irregular vertical ridges. Although the Colombian specimens and *S*. *smithwoodwardi* teeth seem to be very close in morphology they are most likely different taxa, as they are separated by at least ∼50 million years; moreover, they were also separated by a great geographical distance.

## Discussion

The fossil record of Mesozoic chondrichthyans from the northernmost part of South America is scarce and mostly represented by lamniforms and ptychodonts remains from Colombia (Reinhart, 1951; Brito & Janvier, 2002; Carrillo-Briceño et al., 2016; Carrillo-Briceño, Parra & Luque, 2019; Niño Garcia, Parra-Mosquera & Macias-Villarraga, 2019) and Venezuela (Moody & Maisey, 1994; Carrillo-Briceño et al., 2008; Carrillo-Briceño, 2009; Carrillo-Briceño, 2012; Carrillo-Briceño & Spencer, 2013; Guinot & Carrillo-Briceño, 2018), although a single report of a hybodontiform shark based on an isolated dorsal fin spine was also reported for the Rosa Blanca Fm. (Carrillo-Briceño et al., 2016). Hybodontiform reports in South America are also scarce, with some articulated and semi-articulated specimens of *Tribodus limae* (Brito & Ferreira, 1989), from the Lower Cretaceous of Brazil, and isolated teeth and cephalic and dorsal fin spines, from the Triassic and Jurassic of Argentina (Cione et al., 2002; Johns, Albanesi & Voldman, 2014), Upper Jurassic and Lower Cretaceous of Brazil (Woodward, 1888; Pinheiro et al., 2011; Pinheiro et al., 2013; Silva et al., 2011; Cupello et al., 2012), and Upper Jurassic-?Lower Cretaceous of Uruguay (Soto, Perea & Toriño, 2012). In this case, *Strophodus rebecae* sp. nov., from the Lower Cretaceous of Colombia represents the first report for the genus in the Americas. Previously, Bryant (1914) described *Strophodus shastensis* from the Upper Triassic of California in North America, which later was assigned to *Asteracanthus shastensis* by Jordan & Hannibal (1923); nevertheless, this species is currently recognized within the genus *Palaeobates* (Meyer, 1849) (Cuny, Rieppel & Sander, 2001; Cappetta, 2012; Pla, Márquez-Aliaga & Botella, 2013). Other reports of *Asteracanthus* from North America are restricted only to isolated fin and cephalic spines (*e.g.*, Leidy, 1873; Miller, 1968; Baird & Horner, 1979).

As previously referenced in the differential diagnosis section, anterolateral and lateral teeth of *Strophodus rebecae* sp. nov., can be differentiated from at least 12 *Strophodus* species recognized from the Jurassic of Asia and Europe (Agassiz, 1838; Peyer, 1946; Goto, Kuga & Hachiya, 1991; Michelis et al., 1996; Rees & Underwood, 2008; Citton et al., 2019; Szabó & Főzy, 2020; Kumar et al., 2022; Sharma & Singh, 2021; Stumpf, Meng & Kriwet, 2022, and references therein). In reference to ?*Asteracanthus biformatus* (Kriwet, 1995) from the Upper Jurassic of Portugal, this species was described based on cephalic and fin spines, though an isolated tooth was reported as ?*A*. *biformatus* (Kriwet, 1995, plate 1, fig 3). Stumpf et al. (2021) questions the validity of *A. biformatus*, like that of other described species that were identified based only on isolated fin spines due to the lack of discrete morphological characters for use in species differentiation. Szabó & Főzy (2020) also considered *A*. *biformatus* as a nomen dubium due to the poor preservational condition of the tooth assigend to this species. We share the opinion of Szabó & Főzy (2020) with respect to the bad preservational conditions of the isolated tooth of *A*. *biformatus*. However, the possibility that this tooth could belong to *Strophodus* should not be ruled out, and future work on that material or future findings could offer new insights on this. We also agree with the opinion of Szabó & Főzy (2020) regarding the validity of *Strophodus normanianus* (Dollfus, 1863), and the validity as comparative material of *Asteracanthus* (?*Strophodus*) *somaensis* Yabe, 1902, from Japan due to poor illustrations of the material. Although here we do not present a descriptive comparison with teeth referred to open nomenclature, our specimens from Colombia, especially lateral teeth, look different to those of indeterminate *Strophodus* species from the Jurassic of Madagascar (Priem, 1907), Europe (*e.g.*, Peyer, 1946; Kriwet, Rauhut & Gloy, 1997; Vincent et al., 2013; Wills et al., 2019), and Asia (Goto, Kuga & Hachiya, 1991; Cuny, Suteethorn & Kamha, 2005; Cuny et al., 2009). Although the lateral tooth of *Strophodus* sp. illustrated by Cuny, Suteethorn & Kamha (2005, fig. 4), from the Jurassic of Thailand could resemble in in occlusal outline the lateral teeth of *Strophodus rebecae* sp. nov. Additionally, there is an unnamed species of *Strophodus* from the Tithonian of Germany, a specimen represented possibly by the most complete an associated set of jaws with teeth, which was described by Pfeil (2011) as *Asteracanthus* sp. The anterolateral and lateral teeth of this unnamed *Asteracanthus* species appear to be similar in shape (occlusal outline) to the teeth of *Strophodus rebecae* sp. nov. (see Pfeil, 2011, figs. 7–8). However, it can be noticed that lateral teeth in *Strophodus rebecae* sp. nov. have more concave lingual edges, while in the specimen from the Tithonian of Germany the lingual edges in lateral teeth seem to be straight (Pfeil, 2011, figs. 7–8). Better preparation and future detailed descriptions in the specimen from the Tithonian of Germany might be necessary to define the diagnostic characters of that unnamed species. It is important to note that Pfeil (2011) regarded the monospecific genus *Bdellodus* Quenstedt, 1882, from the Toarcian of Germany, as a junior synonym of *Strophodus (= Asteracanthus)*. In our opinion, this assumption suggested by Pfeil (2011) should be taken with caution, this is because the teeth of *Bdellodus bollensis* Quenstedt, 1882, seem to have a morphology and a dental pattern (specially in ?lateral teeth) completely different from that observed in *Strophodus*.

In reference to Cretaceous reports of *Strophodus* (= *Asteracanthus* sp.), its record is relatively scarce in comparison to those from the Jurassic (*e.g.*, Yabe, 1902; Priem, 1907; Peyer, 1946; Goto, Kuga & Hachiya, 1991; Goto, Uyeno & Yabumoto, 1996; Cuny, Suteethorn & Kamha, 2005; Cuny et al., 2007; Cuny et al., 2009; Cuny et al., 2014; Rees & Underwood, 2008; Vincent et al., 2013; Rigal & Cuny, 2016; Romano et al., 2018; Wills et al., 2019; Szabó & Főzy, 2020; Kumar et al., 2022; Sharma & Singh, 2021), and references therein). Most of the *Strophodus* records from the Cretaceous have been reported as indeterminate species from the Valanginian-Albian range of Europe, including England (Batchelor & Ward, 1990), France (Priem, 1912; Guinot, Cappetta & Adnet, 2014), and Switzerland (Pictet & Campiche, 1858; Peyer, 1946), and these teeth look different to those of *Strophodus rebecae* sp. nov. Recently Sokolskyi & Guinot (2021) suggest a pending taxonomic status for *Asteracanthus* (= *Sphaenonchus*) *compressus* Rogovich, 1860, from Albian deposits of Ukrainian due to the disappearance of the holotype. The only existing evidence of *A*. (= *Sphaenonchus*) *compressus* is illustrated in Rogovich (1860, pl. 1, figs. 9–10), which shows what appear to be tooth fragments and the base of a cephalic spine. Sokolskyi & Guinot (2021) considered this species name as valid pending further sampling and/or information on the whereabouts of the missing specimen, which would be essential to clarify the taxonomy of this dubious species. *Asteracanthus aegyptiacus* Stromer, 1927, from the Late Cretaceous of Africa, was described based on isolated dorsal and cephalic spines; but recently, Michaut (2017), using a great number of isolated fin and cephalic spines and teeth from the Maastrichtian of Niger, attempted to reconstruct the dentition of this species. However, the assignment of this material to *Asteracanthus*, or even *Strophodus* should be taken with caution, because the dental morphology of *A. aegyptiacus* seems to be very different from the two above mentioned taxa. Batchelor & Ward (1990) had already suggested that *A. aegyptiacus* has teeth that differ significantly from those of *Asteracanthus* and *Strophodus*. Recently Stumpf et al. (2021) noticed that *A. aegyptiacus* exhibits a unique ornamentation pattern in its spines (see Stromer, 1927; Cappetta, 1972; Werner, 1989), that differs from that observed in stratigraphically older fin spines referred to *Asteracanthus*, thus making a positive referral to the later genus unlikely. According to Stumpf et al. (2021) *A*. *aegyptiacus* spines co-occur with those attributed to *Hybodus* as well as teeth of distobatid taxa. This could suggest that *A*. *aegyptiacus* could be congeneric with one of the co-occurring distobatid species of the Late Cretaceous of Africa, for example *Distobatus* Werner, 1989, and *Aegyptobatus* Werner, 1989. Taking into consideration all of this mentioned above, *Strophodus rebecae* sp. nov. could be tentatively considered the only valid Cretaceous species and the only record of the genus from Gondwana at the time. The known reports of *Strophodus* from the Cretaceous of Europe and South America suggest tentatively that the youngest record of the genus in both continents could be ?Albian in age (see Pictet & Campiche, 1858; Priem, 1912; Peyer, 1946; Batchelor & Ward, 1990; Guinot, Cappetta & Adnet, 2014).

Two fragmented and non-diagnostic lateral teeth from the Rosa Blanca Fm. are tentatively assigend here to *Strophodus* sp. (UR-CP-0132, Figs. 2H1–2H4, and UR-CP-0134, Figs. 2G1–2G2, from La Virgen West and El Sapo South localities, respectively). Although these specimens could belong to *Strophodus rebecae* sp. nov., their poor state of preservation does not allow us to diagnose these specimens. *Strophodus rebecae* sp. nov., corresponds to the second record of a hybodont shark from the Rosa Blanca Fm., since two large dorsal fin spines of an indeterminate species of ?*Asteracanthus* were reported by Carrillo-Briceño et al. (2016) from another locality (El Alto) different from where the specimens describe here come. An accurate taxonomical assignation for these two isolated dorsal fin spines is not possible due to their poor state of preservation. However, since *Strophodus* most likely had tuberculate fin spines (Stumpf et al., 2021), the material described by Carrillo-Briceño et al. (2016) combined with the *Strophodus* teeth described here, might indicate the presence of at least two distinct hybodontiforms in the Rosa Blanca Fm.

The presence of *Strophodus rebecae* sp. nov. increases the paleodiversity known for this geological unit. Other vertebrates reported for the Rosa Blanca Fm., particularly from the surrounding areas of the Zapatoca town, include indeterminate fish remains (Cadena, 2011a; Benavides-Cabra & Páramo-Fonseca, 2020), turtles (Cadena & Gaffney, 2005; Cadena, 2011a; Cadena, 2011b; Cadena, 2020; Cadena, Jaramillo & Bloch, 2013), pterosaurs (Cadena, Unwin & Martill, 2020), ichthyosaurs and elasmosaurid remains (Cadena, 2011a; Benavides-Cabra & Páramo-Fonseca, 2020), and reported but not fully described material of a metriorhynchoid crocodylomorph (Larsson, 2012). Abundant dental and cranial remains of indeterminate pycnodont fishes (see Fig. 1D) were collected by one of the authors (E-AC) from the La Virgen West, Pico de la Vieja South, El Caucho, and El Sapo North and South localities of the Rosa Blanca Fm. and are currently under study. The Rosa Blanca Fm. (Figure 3) has been interpreted as shallow marine deposits (Etayo-Serna & Guzmán-Ospitia, 2019; Guzmán-Ospitia, 1985). For this reason, it is not unreasonable to suggest that *Strophodus rebecae* sp. nov., was a species adapted to purely marine conditions, as has been reported for the other species of the genus, although freshwater-influenced isotopic composition was found in some *Strophodus* teeth of the Jurassic of Switzerland (Leuzinger et al., 2015). The trophic structure of the Central Atlantic Ocean at the northernmost portion of South America during the Valanginian and Hauterivian is still poorly known. Durophagous hybodontiform sharks, as well as other shell-crushing fishes like pycnodontiforms, must have played an important ecological role as predators in these ancient tropical coastal ecosystems, due to the abundant and diverse invertebrate fauna present at the Carrizal and El Sapo members, similar to proposals for other Cretaceous and Jurassic faunas (Kriwet, 2001; Cawley et al., 2021; Cooper & Martill, 2020).

## Concluding remarks

*Strophodus rebecae* sp. nov. from the Valanginian-Hauterivian of Colombia undoubtedly represents the only Cretaceous record of the genus from Gondwana, filling a gap in the knowledge of the paleodiversity and geographic distribution of this group of hybodontiforms outside of Europe during the Cretaceous. Additionally, this new record offers new insights into the scarce hybodontiforms paleodiversity known from South America. Paleoenvironmental inferences from the Rosa Blanca Fm. suggest that this species was adapted to marine environments, with a typical crushing-type dentition that may have allowed it to play an important role as a durophagous predator in this ancient tropical coastal ecosystem. The known fossil record of *Strophodus* from the Cretaceous of Europe and South America suggests that the youngest record of the genus could be ?Albian in age.

## Institutional abbreviations

UR-CP: Paleontological collection, Facultad de Ciencias Naturales, Universidad del Rosario, Bogotá, Colombia

## Additional Information and Declarations

### Field Study Permissions

The following information was supplied relating to field study approvals (i.e., approving body and any reference numbers): Field permits were granted by the Servicio Geológico Colombiano to Edwin Cadena via radicado No. 20203800077871. Landowner’s I. Díaz and F. Serrano allowed us to collect some of the fossils in their properties (permit obtained via personal communication).

### Data Availability

The following information was supplied regarding data availability: All data is included in the article and figures.

### New Species Registration

The following information was supplied regarding the registration of a newly described species: Publication LSID: urn:lsid:zoobank.org:pub:11ED37F2-6168-41CB-8DF3-AAE56976D9D6 Species name: urn:lsid:zoobank.org:act:16C57BBE-99F8-48E5-8A00-B172A09353DD
